# Predicting Bankruptcy in Wholesale, Retail, and Motor Vehicle Repair: An AI-ML Perspective

**DOI:** 10.12688/f1000research.170279.1

**Published:** 2025-11-14

**Authors:** Nagaraju Thota, Guruprasad Desai, Sreenivasulu Puli, A.C.V. Subrahmanyam, V N Vishweswarsastry

**Affiliations:** 1 Department of Economics and Finance, Birla Institute of Technology & Science Pilani - Hyderabad Campus, Hyderabad, Telangana, 500078, India; 2 Manipal Academy of Higher Education, Manipal, Karnataka, India

**Keywords:** Bankruptcy Prediction; AI-ML Models, Trade Services Sector, SMOTE, Early Warning Indicators, Information Value.

## Abstract

**Background:**

Bankruptcy prediction is crucial for financial stability, and sector-specific Artificial Intelligence and Machine Learning (AI-ML) models have proven superior in performance. However, a significant gap exists, as most models are designed for advanced economies, leaving their efficacy in emerging markets like India unexplored. This study addresses this gap by focusing on the applicability of these advanced models to predict bankruptcy within India’s dynamic trade services sector.

**Methods:**

The research utilized a substantial sample of 5,527 Indian companies. To counter the challenge of having far fewer bankrupt firms than solvent ones, the Synthetic Minority Oversampling Technique (SMOTE) was employed. The study then leveraged a comprehensive suite of eight popular AI-ML models, including Random Forests, Gradient Boosting, Neural Networks, and Support Vector Machines. To add practical context, business rules based on key financial metrics—liquidity, profitability, and asset size—were integrated.

**Results:**

The findings robustly demonstrate that AI-ML models can accurately predict bankruptcy in Indian trade services firms. A critical discovery was the variation in early warning signals between an analysis of the entire dataset (aggregate) and segmented groups of companies. This indicates that a one-size-fits-all approach obscures important, segment-specific risk factors. The segmented analysis successfully uncovered hidden risks that were not apparent at the aggregate level.

**Conclusions:**

The study concludes that AI-ML models are highly effective for bankruptcy prediction in India’s trade services sector. For stakeholders like investors and creditors, the key takeaway is the superior value of a segmented analytical approach. This strategy maintains high predictive accuracy while revealing nuanced, specific risks. Ultimately, it provides a powerful, tailored tool for safeguarding financial interests in an emerging market context.

## 1. Introduction

It is well established that trade is the lynchpin on which the global economy rests. In its most fundamental sense, trade enables transfer of factors of production across the globe enabling value realization and growth. While the above connotation is usually referred to in the context of international trade, within the geographical boundaries of countries, the internal commerce run through the wholesale and retail trade firms assume a pivotal role of connecting consumers and producers across the value chain ( Buele et al., 2021 **)**. It is also observed that the retail trade sector brings innovation and competitive prices to the consumers (ibid). Using intra-state trade data, it is observed that in India, regional trade is significantly correlated with is manufacturing prowess and has a positive correlation with the income of the regions. Besides, India’s internal trade is estimated to be 1.7 times its international trade ^1^.

Further, the general focus of bankruptcy studies has been on manufacturing companies and the financial institutions in the services sector. Notwithstanding the crucial role played by such sectors in the economy, the trade service sector also has an important role in the internal trade of the country. They also provide both direct and indirect employment to large volumes of casual and skilled labor in the Indian case. As per retail trade industry report, the contribution of the retail trade sector to India’s GDP stood at 10 per cent and its share in employment is around 8 percent ^2^. Further, as at the end of March 2023, the trade sector accounts for 8 per cent of the bank borrowers and close to 10 per cent of the outstanding bank credit in India ^3^. The Indian retail sector is expected to reach a size of USD 2 trillion dollars by 2032 by value ( ASSOCHAM, 2021), thus becoming a crucial link in the aspiration to become a high-income economy. These facets establish that the trade service sector accounts for a significant part of the bank credit and economic activity.

Hence, in this research study, we explore the analytical framework using various AI-ML methods to predict the bankruptcy incidence in the ‘wholesale trade, retail trade and repair of motor vehicles sector’ in India. The analysis is pertinent on two counts. First, it is observed in the literature that industry-specific features impact bankruptcies and resultantly, there is a need to curate the AI-ML models at a sectoral level to achieve a stable performance ( Agrawal and Maheswari, 2019). Second, trade service sector ^4^ has witnessed a fair share of bankruptcies in the Indian context (around 241 companies, approximately 15 percent of sample observations). Hence, it is important to understand the nature of bankruptcies in this segment and benchmark the performance of the AI-ML models in predicting bankruptcies in this sector. Further, the application of AI-ML models provides the stakeholders with tools and techniques not only to assess the bankruptcy risks but also track the key variables as early warning indicators to initiate corrective actions.

Accordingly, the analytical frameworks like the ones used for testing the effectiveness of AI-ML models in predicting bankruptcies in various sectors employed in the literature are extended to the trade service sector. Albeit some caveats follow. The data for the trade service sector is not completely homogeneous as it contains data on wholesale firms, retail firms and repair of motor vehicles. While the granular sub-sector identification is not possible given the data constraints, the analytical framework of using standard AI-ML models is still relevant and useful as it is expected to generate predictions which can provide guidance on bankruptcy risks in this sector. Bekkar et al. (2013) a set of combined measures and graphical performance assessments to provide a more credible evaluation for imbalanced data learning. Also, the application of business rules to provide finer insights needs to be curated for the trade service sector as its nature significantly differs from other major sectors such as manufacturing and construction firms. The rest of the paper is organized into four sections. The second section provides a brief literature review given the paucity of the studies in the specific domain. The third section details the data and methodological framework of the study. The results and concluding observations are presented in the fourth and fifth sections respectively.

## 2. Literature review

Despite their prominent role, only a few studies have dedicated a review or applied AI-ML models for bankruptcy prediction in trade service sector. A brief survey of the literature in chronological order is presented here in chronological order. Using publicly available information of Croatian companies, Pervan et al. (2011) examined the Croatian manufacturing and trade/wholesale company’s bankruptcy and concluded that logistic regression predicts better than the discriminant analysis due to the presence of non-normality features in the data. Němec and Pavlík (2016) tried to predict the insolvency risk of the Czech companies using the balance information of various industries along with the wholesale and retail trade; repair of motor vehicles and motorcycles by employing various methodologies and concluded that multivariate logit has produced the 84 percent accurate results compared to other methodologies. In the case of Greek, Arnis (2018) found that among the bankruptcy prediction models, probit has the highest predictive power and among the variables, debt burden (i.e. loan capital to total funds) is very useful variable in the predicting the Greek company’s bankruptcy in particular the manufacturing industry, wholesale, retail and service sectors.

Though Mackevičius et al. (2018) did not empirically examine the bankruptcy prediction in Lithuania but highlighted the need for an early bankruptcy prediction system for the Lithuania economy due to the rising bankruptcy rates in general across the industries and in particular in the wholesale, retail trade sector. Sourcing the data from SABA database (a popular database in the Europe), Alfaro et al. (2018) examined the Spanish companies bankruptcies using the ensemble methods and concluded that AdaBoost is superior in separating the bankrupt companies from the healthy companies compared to linear discriminant analysis and neural networks in the case of the Spanish wholesale and retail trade; repair of motor vehicles and motorcycles industry. Tanaka et al. (2019) concluded that although random forest achieved the highest bankruptcy prediction accuracy across various industries in OECD countries, including wholesale and retail trade, and repair of motor vehicles and motorcycles, the top five predicting variables varied among the industries.

Matsumaru et al. (2019) examined the bankruptcy prediction using all the listed firms in Japan and concluded that support vector machine technique predicts the bankruptcy more accurately than the multi discriminatory analysis and artificial neural networks at the aggregate level as well as at the individual industry level. Using a sample of 23 bankrupt and 30 healthy trade industry (i.e. wholesale) companies from the western European countries, Vuković et al. (2020) found five key predictors such as ROE, current assets/total assets, solvency, working capital turnover, stocks/current assets. Bogdan et al. (2021) examined bankruptcy of Croatian companies from various industries (around 25 percent firms are from wholesale and retail trade; repair of motor vehicles and motorcycles) using the multiple discriminant analysis (MDA) and logistic regression (logit) methodologies and found that logit model outperformed the MDA in predicting the bankruptcy across the industries in Croatia.

Puli et al. (2024) study contributes to the literature by developing a robust early warning system for India, employing a suite of AI-ML models to predict periods of banking fragility. The findings demonstrate the superior predictive capability of techniques like neural networks and random forests, while identifying credit, interest rate, and liquidity variables as the most critical early warning indicators.

Using the Altman Z-Score methodology, Buele et al. (2021) examined the probability of the failure of a 102 wholesale and retail trade companies of Azuay province of Ecuador and concluded that 49 percent of these companies are in safe zone, 43 percent of them are in gray zone and only 8 percent of them are in danger zone. By employing a double stochastic Poisson model on Poland’s public and non-public companies, Berent and Rejman (2021) achieved around 85 percent of default probabilities of various industries including the wholesale and retail trade; repair of motor vehicles and motorcycles.

From the literature review, it’s evident that there are very few studies that have focused on the “wholesale and retail trade; repair of motor vehicles and motorcycles” sector, and none of them are from India. Furthermore, only a couple of studies ( Matsumaru et al., 2019; Tanaka et al., 2019) have examined advanced countries, with the majority being from Europe. With this in view, this study aims to fill the literature gap, especially from the perspective of emerging countries like India.

## 3. Data and methodology

### 3.1 Data

The list of (241) bankrupt companies in the “wholesale trade, retail trade, and repair of motor” sector is sourced from the Insolvency and Bankruptcy Board of India. The aim of the study is to predict or label a company as bankrupt or otherwise given the financial data of the company. This fits the description of the classification problem, which can be addressed using AI-ML models ( Pompe and Feelders, 1997). However, to deploy AI-ML models, the training dataset should contain adequate representations from both positive and negative classes, viz., bankrupt and non-bankrupt companies. The efficacy of AI-ML models to predict bankruptcy risks in the trade services sector a sample comprising 5527 firms from wholesale trade, retail trade, and repair of motor vehicles is considered due to data availability ( Table 1). Of these 5527 firms, 241 were bankrupt. Hence, to achieve a balanced dataset, SMOTE technique is used to create an oversample dataset comprising 5286 functional and 5286 bankrupt firms.

**Table 1.** Sector wise number of bankrupt and non-bankrupt firms.

Sector	Listed		Non-listed		Grand Total
	Non-bankrupt	Bankrupt	Non-bankrupt	Bankrupt	
A - Agriculture, forestry and fishing	49	6	389	36	480
B - Mining and quarrying	35	3	181	6	225
C - Manufacturing	303	136	329	487	1255
D - Electricity, gas, steam and air conditioning supply	21	3	648	41	713
E - Water supply; sewerage, waste management and remediation activities	0	0	11	0	11
F - Construction	21	35	138	124	318
G - Wholesale and retail trade; repair of motor vehicles and motorcycles	774	34	4512	207	5527
H - Transportation and storage	73	7	849	39	968
I - Accommodation and Food service activities	62	3	408	19	492
J - Information and communication	272	14	1442	33	1761
K - Financial and insurance activities	748	37	2732	103	3620
L - Real estate activities	0	0	6	0	6
M - Professional, scientific and technical activities	74	6	719	21	820
N - Administrative and support service activities	121	8	1237	30	1396
O - Public administration and defence; compulsory social security	2	0	36	0	38
P - Education	19	2	119	4	144
Q - Human health and social work activities	44	2	311	12	369
R - Arts, entertainment and recreation	7	0	39	4	50
S - Other service activities	8	1	72	1	82
- Others	76	8	392	20	496
Grand Total	2709	305	14570	1187	18771
	3014		15757		18771

Source: NACE (Nomenclature of Economic Activities) Key words, IBBI and CMIE Prowess.

The share of bankrupt to non-bankrupt companies is around 50:50 resulting in a dataset that is balanced on both positive and negative classes. This addresses the class imbalance issue which affects the efficacy and accuracy of the AI-ML models dealing with the classification problem ^5^. The set of explanatory variables used in this study are given Table 2, they include firm level financial variables and ratios drawn from similar studies in the domain of bankruptcy prediction. Further, we have also tried to predict the bankruptcy in the trade sector by dividing the sample on the basis of different business rules (liquidity, profitability, and firm asset size).

**Table 2.** List of financial ratios/variables used as explanatory variables.

S. No	Financial ratio/variable	Notation used
1	Profit before interest and taxes to interest expenses	PBIT_INT
2	Cash flows to debt	CF_D
3	Debt to total asses	D_TA
4	Return on assets	ROA
5	Profit margin	PMN
6	Profit after tax to total assets	PAT_TA
7	Quick ratio	QR
8	Sales to working capital	S_WC
9	Profit before interest and taxes to sales	PBIT_S
10	Current ratio	CR
11	Working capital to total assets	WC_TA
12	Cash flows to total assets	CF_TA
13	Asset turnover	ATR
14	Asset growth	AGR
15	Cash flows to sales	CF_S
16	Sales to total assets	S_TA
17	Revenue growth	RGR
18	Total loans to total assets	TL_TA
19	Profit growth	PGR
20	Retained profit growth rate	RPGR

Source: Author Own calculation.

### 3.2 Methodology - AI-ML models

Generally using the labelled data, the supervisory machine learning models extracts the patterns from the training dataset. Further, supervisory machine learning is divided into two categories of algorithms: regression based and classification-based approaches. Regression based supervisory machine learning approach is used to predict the continuous variables whereas the classification based supervisory machine learning approach is used to predict the dichotomous/categorical variables. In this study, our interest variable is dichotomous in nature. Therefore, we focus on the classification type of supervisory machine learning approaches which are very popular in the bankruptcy prediction literature are Logistic Regression (LR), Random Forests (RF), Naïve Baye (NB), Gradient Boosting (GB), Support Vector Machines (SVM), K-Nearest Neighbours (KNN), Decision Trees (DT), and one popular artificial intelligence technique such as Artificial Neural Networks (ANN or NN). Though, many algorithms are available within the supervisory machine learning category, we employ the aforementioned 8 AI-ML techniques on the basis of their popularity in the bankruptcy prediction literature and their explainability, training and prediction speed and ease of implementation.

Further, in this study, we have chosen to test the efficiency of these 8 AI-ML models, slightly departing from the practice adopted in the literature, wherein the focus is on using a single technique or a couple of techniques. Alaka et al. (2018) did a systematic review of 49 articles for the use of AI-ML models for bankruptcy prediction. The authors note that, of the 49 studies under review, only 30 studies compared the performance of the bankruptcy predictions by the AI-ML models. Further, a few techniques viz., Support Vector Machines, Artificial Neural Networks, are compared more often than others (ibid). Contrary to this, the present study has consistently used the 8 AI-ML models viz., LR, RF, NB, GB, SVM, KNN, DT, and NNs, for bankruptcy predictions in the Indian case. The literature on the use of AI-ML models for classification problems in general and bankruptcy predictions in particular clearly underscores that no single model outperforms others ( Kumar and Ravi, 2007; Alaka et al., 2018; and Tanaka et al., 2019). Specifically, with reference to the bankruptcy prediction the performance of the models is found to be influenced by sample size, multicollinearity, underlying statistical distributions, computational ability etc.

All the aforementioned models have relative strengths and weaknesses stemming from the underlying data and model requirements. Hence, to alleviate the issue relating to data all the models are tested on a single sample to compare the relative performance. While the chosen sample may be inherently favourable for certain models, given the fact that all models face similar training and testing conditions, the results can be fairly compared. Furthermore, the AI-ML models inherently present a trade-off between the result accuracy and transparency, with models like LR and DT offering better transparency than SVM and NN which have higher accuracy. Hence, to be agnostic to the choice between transparency and accuracy, the analytical framework of this study presents the performance metrics for the chosen 8 AI-ML models coherently, leaving the researcher or practitioner to make his or her choice based on the use-case at hand. Given the widespread use of these models in the literature, we omit technical details for the sake of brevity.

### 3.3 Performance metrics

Literature establishes that the performance of classification models is evaluated through the construction of confusion matrices ( Kuhn and Johnson, 2013). These matrices are a cross tabulation of number of actual cases and predicted cases as given below. In general, the positive class refers to the variable of interest. In this case “crisis” period is a positive class, with “non-crisis” period being a negative class. The confusion matrices ( Table 3) are then used for computing metrics that enable comparison of the model performance.

**Table 3.** Confusion matrix.

Number of Instances		Actual	
		Positive	Negative
Predicted	Positive	True Positives (TP)	False Positives (FP)
	Negative	False Negatives (FN)	True Negatives (TN)

Source: Author’s own calculation.

Accuracy is the primary metric for assessing AI-ML model performance in classification problems, representing the ratio of correct predictions to total instances. However, it doesn’t account for misclassification errors. To address this, metrics like precision, sensitivity (recall), and specificity are used in Table 4. Precision measures the rate of true positive predictions out of all positive predictions, indicating the model’s ability to avoid false positives. Sensitivity (recall) captures the rate of true positive predictions out of all actual positives, indicating the model’s ability to identify positive instances accurately. The F1-score, the harmonic mean of precision and recall, balances these errors. Specificity measures the rate of true negative predictions out of all actual negatives, akin to sensitivity but for negative instances. AUROC (Area Under Receiver Operating Characteristic Curve) assesses the model’s accuracy in distinguishing between positive and negative classes by plotting sensitivity against 1-specificity. A higher AUROC indicates better model performance. These metrics provide a comprehensive evaluation of AI-ML models beyond simple accuracy.

**Table 4.** Model performance metrics.

Test metric	Specification
Accuracy	TP+TN/ (TP+FP+FN+TN)
	Total correct predictions/
	Total instances in the dataset
Precision	TP/ (TP+FP)
	Correct positive predictions/
	Total positive predictions
Recall	TP/ (TP+FN)
(Sensitivity)	Correct positive predictions/
	Total positive instances
Specificity	TN/(TN+FP)
	Correct negative predictions/
	Total negative instances
F1- Score	2*(Precision*Recall)/ (Precision +Recall)
	Harmonic mean of precision and recall

Source: Author’s own calculation.

### 3.4 Application of business rules business overlay

Although AI-ML models provide very good accuracy rates as compared to traditional econometric models, they fail to provide a convincing causative link between explanatory variables and the predicted variables. One of the key concerns using AI-ML models is that the models often function as a black box, wherein only inputs and outputs are visible to the user ( Guidotti et al., 2018). While some AI-ML models do provide some guidance regarding causation they fall short of establishing a formal relationship between explanatory and predicted variables ( Freitas, 2014). To this end, to improve the explainability of the models used, this study applies business rules to add context to the predictions made by the AI-ML models. Though this falls short of providing a definitive causal link, it can provide direction of likely impact on the predicted variable given the business rules. Also, financial regulators often stipulate dispensations to mitigate stressed firms to avoid bankruptcy or failure based on differential criteria regarding asset size, profitability, and liquidity positions etc. This allows the regulators to ensure that benefits of such dispensations are utilized by genuine firms under stress and avoid a one-size fits all approach ( RBI, 2020, 2023) ^6^. Hence, these business rules are framed using the conventional credit risk or investment analysis used by banks and fund houses for selecting or monitoring their investments. The study uses the following three business rules based on liquidity, profitability, and asset size position of the firms.

**3.4.1 Liquidity based business rules**

One of the early warning signs about financial distress in a firm is mismanagement of liquidity, often resulting in default and distress precipitating in bankruptcies. Hence, bankers traditionally stipulate minimum levels of liquidity parameters to be achieved or maintained by the firms to get credit facilities. To illustrate, firms should have quick and current ratios of minimum 1.00 and 1.33 respectively, which signals that the current assets of the firm are adequately covering the current liabilities ( Venkatachalam and Natarajan, 2015). Therefore, bankers and investors are more likely to monitor such liquidity ratios and form an opinion about the firm’s financial health. Hence, the sample data is bifurcated into two sets (A and B) using the liquidity thresholds mentioned above. The firms with quick and current ratio above 1.00 and 1.33 are categorized as firms with healthy liquidity, while those below the liquidity thresholds are categorized as firms with liquidity issues. Subsequently, the AI-ML models are run on samples A (healthy liquidity firms) and B (weak liquidity firms) after removing the liquidity ratios from the explanatory variables. Such an assessment primarily considers the liquidity parameters which are key to decisioning by the banks and investors and then looks at the risks of bankruptcy.

**3.4.2 Profitability based business rules**

Like liquidity ratios, another key early warning indicator that banks and investors look out for monitoring firms is their profitability. Generally, bankers and investors approach firms that are profit making differently from those that are incurring losses in terms of investment strategy. Hence, the sample dataset is bifurcated in two sub-sets (A and B) based on the profitability of the firms viz., profit making (ROA being positive) and loss making (ROA being negative). Subsequently, the AI-ML models are run on samples A (profit making firms) and B (loss making firms) to look out for risks of bankruptcy, beyond profitability.

**3.4.3 Asset size based business rules**

Notwithstanding the profitability and liquidity status of the companies, another key decision parameter considered by banks and investors is the size of the firm i.e., total assets. The selection and application of credit risk techniques vary depending on the size of the firm. Small firms may be highly vulnerable to macro-economic shocks and pose high risks, while large firms can better withstand such risks, their failure can have very high costs for the banker or investor. Also, in the event of bankruptcy, for larger firms it may take longer to realize the fair value of the stranded assets than compared to smaller firms. Hence, it might be rational for a banker or investor to differentially approach the risk posed by small and large firms. Accordingly, the sample is bifurcated into four categories viz., A, B, C and D based on the asset size of the companies as given Table 5 below. Subsequently, the AI-ML models are run on samples A to D to assess the performance of models and explore the role of various explanatory variables on signalling bankruptcy of firms across asset size categories.

**Table 5.** Classification of companies based on the size of the assets.

Asset size condition	Category
Greater than ₹5,000 Crore	A
Between ₹1,000 and ₹5,000 Crore	B
Between ₹200 and ₹1,000 Crore	C
Lesser than ₹200 Crore	D

Source: Author’s own Calculations.

Applying AI-ML models on the bifurcated datasets based on business rules can provides three insights on predicting bankruptcies among manufacturing companies in India. First, it allows the researchers to assess the performance of AI-MLs models of the bifurcated datasets based on business rules and identify the best performing models for each sub-segment. Second, it can identify the key variables to signal the bankruptcy risks beyond the specified business criteria viz., liquidity, profitability, and asset size. Third, it enables discerning not so good companies from the companies that are seemingly good companies on the specified criteria. From an investment risk analysis standpoint, such decision-making insights can be very useful to protect investor interest. The analysis not only offers sharper insights on bankruptcy risks within good performing companies with healthy liquidity and profitability, but also provides a list of variables with high IV values to monitor for picking up the bankruptcy signals. This facilitates investor to apply a differentiated approach to assessing risk across firm categories and better understand business models and risks emanating from them ^7^.

### 3.5 Class imbalance – SMOTE technique

For better bankruptcy prediction, it is crucial that the dataset used for AI-ML models is balanced between positive (bankrupt firms) and negative (non-bankrupt firms) classes. A skewed dataset can lead to higher error rates, as the model may not learn adequately about both classes. While segmenting samples based on business rules offers decision-making insights, it can inadvertently create unbalanced datasets, impacting model performance. To address this, the study employs the Synthetic Minority Oversampling Technique (SMOTE) to generate a balanced dataset ( Kim et al., 2015; Le et al., 2018; Veganzones and Séverin, 2018; Ghatasheh et al., 2020; Smiti and Soui, 2020; Tumpach et al., 2020; Alam et al., 2021; Garcia, 2022; Papíková and Papík, 2022; Amirshahi and Lahmiri, 2024). SMOTE, a widely used data preprocessing method, corrects class imbalances by creating synthetic examples from the minority class based on the feature space rather than the data space ( Fernández et al., 2018). This ensures the AI-ML models are trained on a balanced dataset, improving their predictive accuracy. The study applies AI-ML models to datasets segmented by business rules and balanced using SMOTE, enhancing the model’s ability to predict bankruptcy accurately.

## 4. Results and Discussion

### 4.1 Performance of AI-ML models on full sample

**Figure 1.**
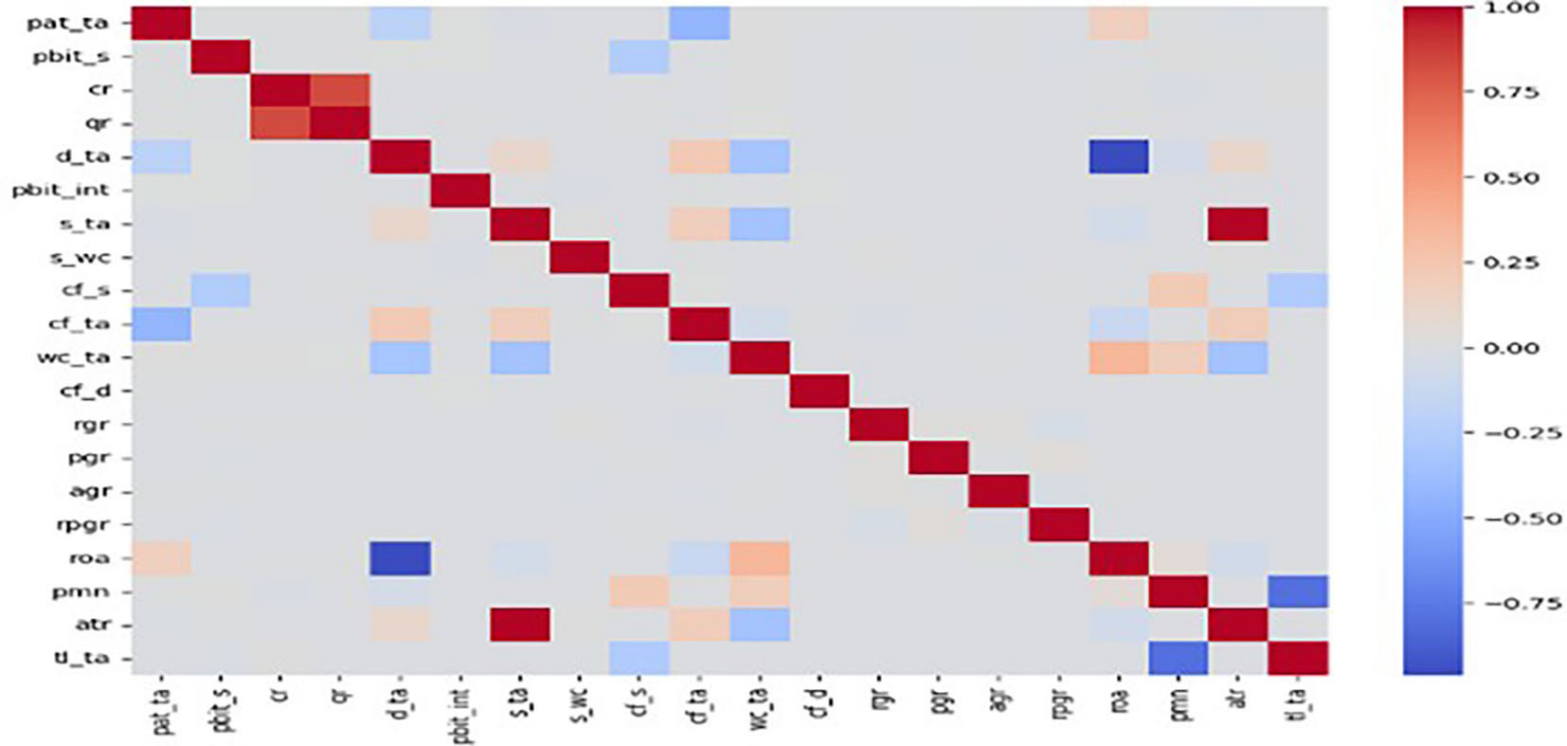
Correlation matrix of numeric features (trade services sector firms).

The efficacy of AI-ML models to predict bankruptcy risks in trade services sector a sample of comprising 5527 firms from wholesale trade, retail trade, and repair of motor vehicle is considered. Of these 5527 firms, 241 were bankrupt. Hence, to achieve a balanced dataset, SMOTE technique is used to create an oversample dataset comprising 5286 functional and 5286 bankrupt firms. Foremost, the correlation matrix of the given in Figure 1 indicates that sparse correlation amongst the explanatory variables, underscoring their usefulness in signalling bankruptcy risks. Subsequently, like in the case of manufacturing and construction firms, we deploy the same 8 AI-ML models on the full sample and followed by the testing on the sub-samples which are bifurcated on the basis of liquidity, profitability, asset size business rules. Further, the testing of AI-ML models in this chapter follows the same methodologies adopted for manufacturing and construction firms. Hence, for brevity, the extended discussions on model performance are not presented in this chapter. The focus is limited to identify key models and the set of explanatory variables with high IV values and the results are presented hereunder.

The performance metrics of the AI-ML models tested on the full sample of firms is given the Table 6. The models boast an average accuracy of around 80 per cent indicating that AI-ML models can be used for predicting bankruptcy risks in the trade services sector. Also, the AUROC scores of models is around 0.83 indicating reasonable discriminatory power of the models. Further, as compared to the performance of AI-ML models for manufacturing and construction firms, the accuracy and discriminatory power of the models is lower in case of trade service firms. However, the performance of random forest and neural network models in case of trade service firms stands out compared to other models. Other models like gradient boosting, k-nearest neighbours, decision trees also register decent performance levels, next only to random forest and neural network models in this sector. Furthermore, the usefulness of the models in discerning both the functional and bankrupt firms is also good. The F1-score for random forest model is 0.96 while that of the neural network model is 0.88 indicating balanced prediction performance.

**Table 6.** Performance metrics of models for predicting bankruptcy considering all the financial factors.

Model	Accuracy	Precision	F1-Score	Sensitivity	Specificity	AUROC
Logistic Regression	0.61	0.61	0.61	0.61	0.62	0.69
Random Forest	0.96	0.96	0.96	0.98	0.94	0.99
Gradient Boosting	0.86	0.87	0.86	0.92	0.8	0.93
SVM	0.67	0.69	0.66	0.51	0.82	0.75
KNN	0.85	0.87	0.85	0.96	0.73	0.93
Decision Tree	0.88	0.88	0.88	0.91	0.85	0.88
Naive Bayes	0.55	0.59	0.48	0.9	0.2	0.6
Neural Network	0.88	0.88	0.88	0.94	0.82	0.94

Source: Author’s own Calculations.

On the basis the information value (IV) or weight of evidence which are available in Table 7, for trade services firms, the top-5 variables with high IV are interest coverage (PBIT_INT), return on assets (ROA), debt to total assets (D_TA), profit to total assets (PAT_TA), and working capital to total assets (WC_TA). The explanatory variables wise, the information value is provided in Table 7 below. The indicators with high IV can perform the role of early warning indicators as they contain relatively higher information about the impending bankruptcy risks than other explanatory variables. Interestingly for trade service firms, the working capital to total assets is a key variable with high IV value. Working capital is more relevant for trading firms as they depend on stock in trade and try to optimize creditors and debtors to maximize their revenues. A typical trade service firm can be thought of as moving stocks in trade, purchasing and/or selling on credit. Such mismatches in sale realizations may necessitate higher working capital requirements.

**Table 7.** Information values of the explanatory variables.

Explanatory variable	IV
PBIT_INT	0.907
ROA	0.864
D_TA	0.681
PAT_TA	0.646
WC_TA	0.635
CR	0.586
PMN	0.502
QR	0.381
PBIT_S	0.327
S_WC	0.23
CF_D	0.22
RGR	0.188
RPGR	0.172
CF_TA	0.109
CF_S	0.103
S_TA	0.085
TL_TA	0.083
ATR	0.083
AGR	0.08
PGR	0.064

Source: Author’s own Calculations.

### 4.2 Performance of AI-ML models on bifurcated sample - Business rules

As observed in case of manufacturing and construction firms, deployment of AI-ML models on the sub-samples bifurcated based on business rules (viz., liquidity, profitability, and asset size) yield interesting insights. Specifically, in terms of IVs of variables, the sub-samples have revealed differential relative impact of explanatory variables to signal bankruptcy risks. Hence, a similar exercise is carried out for the trade service firms. The overall sample is bifurcated into sub-samples using liquidity, profitability, and asset sized based business rules. Further, using SMOTE technique, the sub-samples are balanced. The business rule wise performance metrics of the AI-ML models is presented hereunder.

**4.2.1 Performance of AI-ML models on bifurcated sample (liquidity ratios)**

The performance metrics of the AI-ML models on the sub-samples created using liquidity-based business rules are given in Table 8 and Table 9. The accuracy rates of some of the AI-ML models viz., random forest, gradient boosting, neural network in predicting bankruptcy risks for both firms with and without liquidity problems are above 85 percent. Also, the AUROC scores of these models are above 0.90 indicating strong discriminatory power of the models. The F1-scores of the models are also around 0.90 suggesting a balanced performance of the models. Interestingly, the IVs of the explanatory variables for the companies with and without liquidity issues vary divergently. For companies without liquidity issues, the profit margin, total loans to total assets, asset turnover, debt to total assets, cash flows to sales are the top 5 explanatory variables with higher IV values in Table 10.

**Table 8.** Performance metrics firms above liquidity threshold values of 1.33 and 1.

Model	Accuracy	Precision	F1-Score	Sensitivity	Specificity	AUROC
Logistic Regression	0.67	0.67	0.67	0.64	0.7	0.76
Random Forest	0.99	0.99	0.99	1	0.98	1
Gradient Boosting	0.98	0.98	0.98	1	0.96	0.99
SVM	0.79	0.79	0.79	0.83	0.75	0.86
KNN	0.9	0.91	0.9	0.98	0.81	0.96
Decision Tree	0.96	0.96	0.96	0.98	0.95	0.96
Naive Bayes	0.56	0.66	0.48	0.95	0.17	0.78
Neural Network	0.99	0.99	0.99	1	0.98	1

Source: Author’s own Calculations.

**Table 9.** Performance metrics firms below liquidity threshold values of 1.33 and 1.

Model	Accuracy	Precision	F1-Score	Sensitivity	Specificity	AUROC
Logistic Regression	0.62	0.62	0.61	0.66	0.57	0.68
Random Forest	0.95	0.95	0.95	0.96	0.94	0.99
Gradient Boosting	0.85	0.85	0.85	0.9	0.79	0.93
SVM	0.63	0.66	0.62	0.42	0.84	0.71
KNN	0.82	0.84	0.81	0.95	0.68	0.9
Decision Tree	0.85	0.85	0.85	0.87	0.84	0.85
Naive Bayes	0.54	0.61	0.45	0.94	0.14	0.61
Neural Network	0.83	0.83	0.83	0.87	0.78	0.9

Source: Author’s own Calculations.

**Table 10.** Comparison between various explanatory variables ranked in descending order of IV (IV A - liquidity ratios above threshold, IV B - liquidity ratios below threshold).

Companies with healthy liquidity		Companies with weaker liquidity	
Variable	IV A	Variable	IV B
PMN	0.636	PBIT_INT	0.4184
TL_TA	0.572	PAT_TA	0.3918
ATR	0.5317	RGR	0.3217
D_TA	0.5162	ROA	0.3146
CF_S	0.4465	PBIT_S	0.29
PBIT_S	0.4274	D_TA	0.2135
S_WC	0.4112	PMN	0.1908
S_TA	0.3871	WC_TA	0.1714
ROA	0.3724	RPGR	0.1708
PAT_TA	0.3507	S_TA	0.1255
CF_D	0.2981	ATR	0.1242
PGR	0.29	S_WC	0.1235
CF_TA	0.2829	CF_S	0.1169
AGR	0.2772	CF_D	0.1102
PBIT_INT	0.2491	AGR	0.1077
RGR	0.2151	CF_TA	0.1058
RPGR	0.1936	TL_TA	0.1056
WC_TA	0.1322	PGR	0.1012

Source: Author’s own Calculations.

**4.2.2 Performance of AI-ML models on bifurcated sample (profitability ratios)**

The performance metrics of the AI-ML models on the sub-samples created using profitability-based business rules are given in Table 11 and Table 12. As can be observed from the performance metrics, the average accuracy of the AI-ML models on the sub-samples for profit making and loss-making companies is like that of the overall sample. The average accuracy of AI-MLs for profit making companies is around 84 per cent and for loss making companies the average accuracy is 77 percent. The average AUROC scores of the models are 0.90 for profit making companies and 0.83 for loss making companies. Indicating that AI-ML models have higher discriminatory power to discern bankrupt firms from functional firms in case of profit-making firms than in case of loss-making firms. Also, the average F1-scores of the models follow similar trends between profit- and loss-making firms. Overall, the performance metrics indicate that AI-ML models are performing better in case of profit-making trade service firms than in case of loss-making firms. Notwithstanding the above, the performance metrics of AI-ML models in this case i.e., profitability-based bifurcation is either comparable or better than the levels registered for the overall sample.

**Table 11.** Performance metrics of models for companies which are in profit (ROA > 0).

Model	Accuracy	Precision	F1-Score	Sensitivity	Specificity	AUROC
Logistic Regression	0.72	0.72	0.72	0.76	0.68	0.77
Random Forest	0.98	0.98	0.98	0.99	0.97	1
Gradient Boosting	0.91	0.92	0.91	0.95	0.87	0.97
SVM	0.78	0.81	0.78	0.92	0.65	0.86
KNN	0.88	0.9	0.88	0.98	0.78	0.96
Decision Tree	0.92	0.92	0.92	0.93	0.92	0.92
Naive Bayes	0.55	0.65	0.46	0.95	0.15	0.76
Neural Network	0.95	0.95	0.95	0.98	0.92	0.97

Source: Author’s own Calculations.

**Table 12.** Performance metrics of models for companies which are in loss (ROA < 0).

Model	Accuracy	Precision	F1-Score	Sensitivity	Specificity	AUROC
Logistic Regression	0.66	0.67	0.66	0.76	0.56	0.72
Random Forest	0.95	0.95	0.95	0.96	0.93	0.98
Gradient Boosting	0.87	0.88	0.87	0.92	0.82	0.94
SVM	0.63	0.65	0.62	0.46	0.81	0.75
KNN	0.81	0.84	0.81	0.95	0.68	0.9
Decision Tree	0.87	0.87	0.87	0.91	0.83	0.87
Naive Bayes	0.55	0.61	0.48	0.92	0.18	0.61
Neural Network	0.88	0.89	0.88	0.95	0.81	0.93

Source: Author’s own Calculations.

The IVs of the explanatory variables for the sub-samples based on profitability business rules is given in Table 13. As in the case of liquidity-based bifurcation, in this case too, the variables with high IVs vary for both the profit making and loss-making firms as compared to the overall sample. As observed earlier, for the overall sample, profit margin, return on asset, debt to total asset have highest information content in signalling bankruptcy. Followed by profit after tax to total asset and working capital to total asset. However, for the profit-making firms, the current ratio, interest margin, profit margin, profit to total assets, and working capital to total assets are the top 5 variables with highest IVs. Also, for the loss-making firms, the growth in retained profit, interest coverage, profit before interest and taxes to sales, revenue growth, and profit to total assets are the top 5 variables with highest IVs. It is interesting to note the differences between the set of top 5 variables for the profit- and loss-making firms and with that of the overall sample. While interest coverage and profit to total assets figure out as variables with high IVs for the overall sample, they also figure out in case of both profit- and loss-making firms. Thus, bifurcating the overall sample into sub-samples level provides useful insights.

**Table 13.** Comparison between various explanatory variables ranked in descending order of IV (IV A - companies in profit, IV B - companies in loss).

Profitable companies (ROA Positive)		Profitable companies (ROA Negative)	
Variable	IV A	Variable	IV B
CR	0.7081	RPGR	0.5091
PBIT_INT	0.6174	PBIT_INT	0.4776
PMN	0.5932	PBIT_S	0.4312
PAT_TA	0.5771	RGR	0.4271
WC_TA	0.416	PAT_TA	0.3651
D_TA	0.3972	QR	0.2804
QR	0.3941	CR	0.2752
S_WC	0.3671	WC_TA	0.2574
TL_TA	0.277	D_TA	0.2537
CF_D	0.2057	CF_TA	0.2325
PGR	0.1821	AGR	0.2308
CF_TA	0.1588	S_WC	0.1959
RPGR	0.1416	CF_D	0.1779
ATR	0.1171	TL_TA	0.1721
AGR	0.1098	PGR	0.1539
PBIT_S	0.1089	S_TA	0.135
S_TA	0.1086	ATR	0.1289
CF_S	0.1008	PMN	0.1149
RGR	0.0936	CF_S	0.0777

Source: Author’s own Calculations.

**4.2.3 Performance of AI-ML models on bifurcated sample (Asset size of the company)**

The overall sample is bifurcated into 4 sub-samples based on the asset size of the firms. This enables analysis of the performance of AI-ML models and to glean the relative importance of explanatory variables using IVs in signalling bankruptcy risks across firm sizes. Comparatively the average firm size of trade services firms is lower than that of the manufacturing or construction firms ^8^. The performance metrics of the AI-ML models is given in Table 14, Table 15, Table 16, and Table 17. The IVs of the explanatory variables for the four sub-samples are given in Table 18. From the performance metrics, it can be observed that the average accuracy rate of AI-ML models for categories A, B, C and D companies are at 83 percent, 79 percent, 81 percent, and 84 percent respectively, which is greater than the accuracy rate of 80 percent achieved for the overall sample. Likewise, the AUROC scores for the AI-ML models for category A, B, C and D companies are at 0.90, 0.86, 0.87, and 0.88 respectively as compared to AUROC score of 0.84 achieved for the overall sample. This represents an adequate discriminatory power for the models. Further, across categories of companies, random forest model has achieved accuracy rates of 94 percent to 98 percent and the AUROC scores range from 0.99 to 1.00. Thus, outperforming all other models across categories. Furthermore, neural networks have a high accuracy rate in the case of category D companies.

**Table 14.** Performance metrics of models for companies with category A asset size.

Model	Accuracy	Precision	F1-Score	Sensitivity	Specificity	AUROC
Logistic Regression	0.74	0.74	0.74	0.71	0.78	0.84
Random Forest	0.95	0.95	0.95	0.98	0.91	0.99
Gradient Boosting	0.92	0.92	0.92	0.96	0.88	0.98
SVM	0.8	0.81	0.8	0.86	0.75	0.88
KNN	0.82	0.85	0.82	0.97	0.68	0.89
Decision Tree	0.88	0.88	0.88	0.9	0.85	0.88
Naive Bayes	0.62	0.68	0.59	0.9	0.34	0.78
Neural Network	0.9	0.92	0.9	0.99	0.82	0.95

Source: Author’s own Calculations.

**Table 15.** Performance metrics of models for companies with category B asset size.

Model	Accuracy	Precision	F1-Score	Sensitivity	Specificity	AUROC
Logistic Regression	0.67	0.67	0.67	0.68	0.67	0.74
Random Forest	0.94	0.94	0.94	0.96	0.92	0.99
Gradient Boosting	0.89	0.9	0.89	0.94	0.84	0.96
SVM	0.64	0.66	0.63	0.49	0.8	0.79
KNN	0.83	0.85	0.82	0.96	0.69	0.91
Decision Tree	0.86	0.86	0.86	0.89	0.83	0.86
Naive Bayes	0.58	0.7	0.51	0.96	0.21	0.7
Neural Network	0.89	0.89	0.89	0.95	0.82	0.95

Source: Author’s own Calculations.

**Table 16.** Performance metrics of models for companies with category C asset size.

Model	Accuracy	Precision	F1-Score	Sensitivity	Specificity	AUROC
Logistic Regression	0.7	0.71	0.7	0.61	0.79	0.77
Random Forest	0.96	0.96	0.96	0.98	0.93	0.99
Gradient Boosting	0.91	0.92	0.91	0.96	0.87	0.96
SVM	0.74	0.74	0.74	0.75	0.74	0.82
KNN	0.84	0.86	0.84	0.96	0.72	0.92
Decision Tree	0.89	0.89	0.89	0.91	0.87	0.89
Naive Bayes	0.53	0.6	0.44	0.94	0.12	0.64
Neural Network	0.9	0.9	0.9	0.95	0.85	0.95

Source: Author’s own Calculations.

**Table 17.** Performance metrics of models for companies with category D asset size.

Model	Accuracy	Precision	F1-Score	Sensitivity	Specificity	AUROC
Logistic Regression	0.68	0.68	0.68	0.68	0.68	0.74
Random Forest	0.98	0.99	0.98	1	0.97	1
Gradient Boosting	0.96	0.96	0.96	0.99	0.92	0.99
SVM	0.71	0.73	0.71	0.59	0.83	0.84
KNN	0.9	0.91	0.89	0.98	0.81	0.97
Decision Tree	0.94	0.94	0.94	0.97	0.91	0.94
Naive Bayes	0.57	0.69	0.5	0.96	0.18	0.57
Neural Network	0.97	0.97	0.97	0.99	0.96	0.99

Source: Author’s own Calculations.

**Table 18.** Comparison between various explanatory variables ranked in descending order of IV.

Category A		Category B		Category C		Category D	
Variable	IV A	Variable	IV B	Variable	IV C	Variable	IV D
PAT_TA	0.72	PBIT_INT	0.80	PAT_TA	0.86	PAT_TA	0.86
CR	0.65	ROA	0.75	CR	0.62	PMN	0.64
PBIT_S	0.61	PMN	0.66	PBIT_S	0.59	CR	0.61
PMN	0.52	D_TA	0.64	D_TA	0.58	ROA	0.56
PBIT_INT	0.50	PAT_TA	0.63	PBIT_INT	0.54	RGR	0.42
S_WC	0.50	CF_D	0.40	ROA	0.48	QR	0.40
QR	0.49	RGR	0.39	RGR	0.46	CF_S	0.39
ATR	0.47	CR	0.37	QR	0.46	AGR	0.37
RGR	0.47	AGR	0.36	PMN	0.45	RPGR	0.36
D_TA	0.43	QR	0.35	RPGR	0.44	S_WC	0.36
CF_D	0.40	S_WC	0.32	S_WC	0.38	PBIT_S	0.36
ROA	0.34	PBIT_S	0.31	PGR	0.37	TL_TA	0.35
AGR	0.34	RPGR	0.23	CF_D	0.32	PGR	0.32
PGR	0.30	PGR	0.21	CF_S	0.29	CF_D	0.27
TL_TA	0.24	CF_S	0.16	AGR	0.26	ATR	0.24
CF_S	0.22	ATR	0.14	TL_TA	0.18	D_TA	0.22
RPGR	0.20	TL_TA	0.05	ATR	0.16	PBIT_INT	0.21

Source: Author’s own Calculations.

The analysis of IV of explanatory variables across asset size categories of companies reveals interesting insights in the Table 19. At the overall sample level, interest coverage, return on asset, debt to total asset is seen to be the foremost variables with high IV values. Among these variables, profit to total assets is among the top 5 variables with high IV across all firm types. This is followed by ROA which figures in the top 5 variables for firms in categories B, C, and D, while interest coverage is important for firms in categories A, B, and C. In contrast, debt to total assets is among the top 5 variables only in case of firms in categories B and C. For larger firms (A, B), profit margin is more relevant. While for smaller firms in category D, revenue growth along with profit margin are relevant. Variables like profit before interest and taxes to sales, and current ratio also among the top 5 variables with high IV values. Though there are common variables possessing high IVs both at the overall sample and bifurcated sample, it may be prudent for the investor to adopt a segmented approach to capture the bankruptcy risks in an efficient manner.

**Table 19.** Relative IV of each explanatory variables across asset size categories overall sample.

S. No	Non-bifurcated	Asset size category			
	Full sample	A	B	C	D
1	PBIT_INT	PAT_TA	PBIT_INT	PAT_TA	PAT_TA
2	ROA	CR	ROA	CR	PMN
3	D_TA	PBIT_S	PMN	PBIT_S	CR
4	PAT_TA	PMN	D_TA	D_TA	ROA
5	WC_TA	PBIT_INT	PAT_TA	PBIT_INT	RGR
6	CR	S_WC	CF_D	ROA	QR
7	PMN	QR	RGR	RGR	CF_S
8	QR	ATR	CR	QR	AGR
9	PBIT_S	RGR	AGR	PMN	RPGR
10	S_WC	D_TA	QR	RPGR	S_WC
11	CF_D	CF_D	S_WC	S_WC	PBIT_S
12	RGR	ROA	PBIT_S	PGR	TL_TA
13	RPGR	AGR	RPGR	CF_D	PGR
14	CF_TA	PGR	PGR	CF_S	CF_D
15	CF_S	TL_TA	CF_S	AGR	ATR
16	S_TA	CF_S	ATR	TL_TA	D_TA

Source: Author’s own Calculations.

## 5. Conclusion

The wholesale and retail trade service sector are one of the crucial segments in the economy. This sector has seen its fair share of bankruptcies (247 companies in the sample are from this sector). Hence, the analysis of the AI-ML models to predict bankruptcy risks is extended to this sector on the similar lines carried out for the manufacturing and construction sector. The performance of the AI-ML models at the level of the overall sample is like that of the results obtained in case of manufacturing and construction firms. Albeit the accuracy levels are slightly lower for the firms in the trade services sector. However, the average accuracy and AUROC scores are above 80 per cent and 0.80 representing the usefulness of AI-ML models in predicting bankruptcies in the trade service sector too. An analytical exercise to bifurcate the overall sample into sub-samples based on liquidity, profitability, and asset size-based business rules and test the efficacy of AI-ML models is also carried out for the trade service sector. Based on model accuracy and AUROC scores, random forest model stands out as the best performing model both for the overall sample and sub-samples across business rules. This is followed by neural networks, gradient boosting, and decision tree models.

The interesting facet of the analysis stems from the observations on the information values of the explanatory variables indicating their relative importance to signal bankruptcy risks. The analysis of IVs of the explanatory variables at the level of overall sample indicates that interest coverage, return on assets, debt, profit, and working capital to total assets are the top 5 variables with highest IVs. However, when analysed at the level of sub-samples bifurcate based on business rules, the set of more relevant explanatory variables varies significantly across sub-samples. For firms with liquidity issues, revenue growth is more relevant, while for firms with healthier liquidity profit margin become more important. Similarly, for the profit-making firm’s current ratio and total loans to asset are more relevant contrasting with the loss-making firms where revenue growth and growth in retained profit becomes more important. Also, there are differences in the most relevant variables across firms’ size categories, with profit to total assets figuring out in the top 5 variables across size categories. The results indicate that the investors and stake holders stand to gain from a segmented approach to analyse the bankruptcy risks in using AI-ML models, without losing the predictive accuracy. Further, this approach provides insights on variables with relatively higher information content to signal bankruptcy risks, which may not be visible at an aggregate level.

## Data Availability

The dataset supporting the findings of this study has been deposited in the Figshare repository. To protect participant confidentiality, the data have been de-identified and are available for research purposes only. Figshare: *Dataset for Predicting Bankruptcy in Wholesale, Retail, and Motor Vehicle Repair: An AI-ML Perspective.* Dataset. https://doi.org/10.6084/m9.figshare.30392467 ( Desai, 2025). The project contains the following underlying data:
• Data.xlsx Data are available under the terms of the Creative Commons Attribution 4.0 International license (CC-BY 4.0).

## Footnotes

^1^
Economic survey 2016-17, One Economic India: For Goods and in the Eyes of the Constitution. Government of India.

^2^

Indian Brand Equity Foundation. Retail Industry Report, December 2023. https://www.ibef.org/industry/retail-india

^3^
Basic statistical returns, 2023. Reserve Bank of India.

^4^
For simplicity, henceforth, the wholesale trade, retail trade and repair of motor vehicles together shall be referred to as trade services.

^5^
Class imbalance refers to the situations where either one of the target classes (positive or negative) variables dominates the dataset impacting the training of the algorithm, resulting in higher classification error rates.

^6^
The dispensation allowed by the Reserve Bank of India to mitigated COVID-19 related stress on borrowers adopted a differentiated approach for small and larger firms with criteria specified on financial ratios of firms.

^7^
Although several other business criteria can be applied to understand bankruptcy risks, the present study the attention is limited to the basic and intuitive measures to get across the usefulness of adopting such an analytical framework.

^8^
Reckoning the same, the firms are classified based on asset size into four categories viz., A (>INR 5000 Cr); B (INR 1000 to 5000 Cr); C (INR 200 to 1000 Cr) and D (less than INR 200 Cr)
