# Supplementary material for: Predicting Bankruptcy in Wholesale, Retail, and Motor Vehicle Repair: An AI-ML Perspective
Source: F1000Res. 2026 Jan 13;14:1251. Originally published 2025 Nov 14. [Version 2] doi: 10.12688/f1000research.170279.2 (PMC12831045; doi:10.12688/f1000research.170279.2)
Supplement: Peer review history

# Reviewer report: Reviewer response for version 2

**DOI:** 10.5256/f1000research.194820.r463908

**Published:** 2026-03-12

**Authors:** Dominika Gajdošíková

**Affiliations:** 1 University of Zilina, Slovakia, Slovakia

The paper examines the effectiveness of Artificial Intelligence and Machine Learning (AI-ML) models in predicting corporate bankruptcy within India’s trade services sector. The study is based on a dataset of 5,527 companies, including 241 bankrupt firms, and employs eight machine learning models such as Random Forest, Gradient Boosting, Neural Networks, and Support Vector Machines. To address class imbalance, the authors apply the SMOTE technique and evaluate model performance using repeated k-fold cross-validation. The results suggest that AI-ML models are capable of predicting bankruptcy in the sector with relatively high accuracy, particularly when segmenting firms based on business rules related to liquidity, profitability, and asset size. 

 However, despite these contributions, several areas for improvement were identified that limit the paper’s theoretical grounding, methodological transparency, and analytical clarity.

 1. Although the paper claims to address a gap related to bankruptcy prediction in India’s trade services sector, the originality of the study is not sufficiently articulated. The introduction would benefit from a more precise explanation of how the present research extends existing AI-ML bankruptcy prediction frameworks beyond simply applying established models to a new dataset. The novelty should be clarified in terms of theoretical contribution, methodological innovation, or empirical insight.

 2. The argument that the trade services sector is understudied is presented but not strongly substantiated through systematic comparison with existing studies. A more explicit positioning of this research relative to prior empirical studies would strengthen the originality claim.

 3. The introduction briefly references the importance of trade services and the need for sector-specific models but lacks a comprehensive overview of previous bankruptcy prediction research in both developed and emerging markets. The discussion of foundational studies and recent AI-ML developments should be expanded to better contextualize the research problem.

 4. While the literature review is organized thematically, many references are discussed descriptively rather than critically. There is limited synthesis of how the cited studies collectively inform the research gap. Additionally, the review focuses primarily on methodological developments rather than on sector-specific empirical findings.

 5. Although the study employs eight AI-ML models, the theoretical justification for selecting these specific algorithms is relatively limited. The discussion focuses mainly on their popularity in the literature rather than on their suitability for the specific characteristics of the dataset.

 6. While hyperparameters are listed, the methodological explanation of model training and tuning remains relatively brief. A clearer explanation of feature selection procedures, variable transformations, and potential multicollinearity issues would improve transparency.

 7. The paper introduces twenty financial ratios as explanatory variables but does not sufficiently justify their inclusion based on theoretical or empirical literature. A more structured explanation of how these variables relate to bankruptcy risk would strengthen the methodological framework.

 8. The use of business rules for segmentation (liquidity, profitability, asset size) is interesting but insufficiently justified theoretically. The rationale for the chosen thresholds (e.g., liquidity ratios) should be supported with references or empirical reasoning.

 9. The results are generally presented clearly through performance metrics such as accuracy, precision, recall, F1-score, and AUROC. However, the interpretation of these results remains somewhat descriptive. A deeper discussion explaining why certain models perform better than others would improve the analytical depth.

 10. The practical implications for stakeholders such as investors, creditors, and regulators are mentioned but remain relatively general. The paper would benefit from clearer examples of how the proposed models could be implemented in real-world credit risk assessment or financial monitoring systems. Given the focus on an emerging market context, the study could more explicitly discuss implications for financial regulation, early warning systems, or banking supervision.

Is the work clearly and accurately presented and does it cite the current literature?

Partly

If applicable, is the statistical analysis and its interpretation appropriate?

Partly

Are all the source data underlying the results available to ensure full reproducibility?

Partly

Is the study design appropriate and is the work technically sound?

Partly

Are the conclusions drawn adequately supported by the results?

Yes

Are sufficient details of methods and analysis provided to allow replication by others?

Partly

Reviewer Expertise:

Corporate finance; bankruptcy and financial distress prediction; financial risk assessment; quantitative methods and predictive analytics in economics; machine learning applications in financial modelling.

I confirm that I have read this submission and believe that I have an appropriate level of expertise to confirm that it is of an acceptable scientific standard, however I have significant reservations, as outlined above.

# Reviewer report: Reviewer response for version 2

**DOI:** 10.5256/f1000research.194820.r463907

**Published:** 2026-03-05

**Authors:** Lip Yee Por

**Affiliations:** 1 Universiti Malaya, Kuala Lumpur, Federal Territory of Kuala Lumpur, Malaysia

This manuscript investigates bankruptcy prediction in India’s trade services sector (wholesale, retail, and motor vehicle repair) using a suite of eight AI-ML classifiers (LR, RF, NB, GB, SVM, KNN, DT, ANN), addressing class imbalance via SMOTE, and extending the analysis through business-rule-based segmentation (liquidity, profitability, asset size). The proposed method is interesting but there are several major concerns: 

 1. The manuscript states that 241 bankrupt firms were sourced from IBBI and merged with 5,527 trade-service firms, with a train/test split and cross-validation. However, the time horizon, reference year of financial ratios, and whether the task is one-year-ahead prediction (or contemporaneous classification) are not explicitly defined. Bankruptcy prediction is inherently temporal, and unclear timing can inflate performance if observations are mixed across years.

 Suggestion: Clearly specify (i) the observation period, (ii) how the “bankrupt” label aligns with the financial statement year (s), and (iii) whether the model predicts bankruptcy ahead of time (e.g., t–1 → t). If possible, add a temporal holdout validation (train on earlier periods, test on later periods) and report results alongside random splits.

 2. The paper reports strong performance (e.g., Random Forest/ANN often near the top). Given the original class ratio (241 bankrupt vs 5,286 non-bankrupt), using SMOTE can be appropriate, but it also raises risks of over-optimistic metrics if not handled strictly within folds and with careful model selection.

 Suggestion: Provide a step-by-step, unambiguous description of the pipeline order: split → CV folds → scaling → SMOTE → training → testing. Additionally, report mean ± standard deviation across folds (not just means) and include confidence intervals for AUROC/F1 to demonstrate robustness.

 3. The manuscript lists hyperparameters and states grid search was used. However, it is not entirely clear whether hyperparameter selection occurred inside the repeated cross-validation loop (nested CV) or outside it. If tuning uses information from the same folds used to report performance, results can be biased upward.

 Suggestion: Use nested cross-validation (inner loop for tuning, outer loop for reporting) or clearly state and justify why the current strategy does not leak information. At minimum, provide the tuning search space and confirm that tuning used training folds only.

 4. Segmenting by liquidity/profitability/asset size and ranking variables using Information Value (IV) is practically appealing. However, IV is typically associated with scorecard/logit-style reasoning, and its interpretation alongside tree ensembles and neural networks needs clearer methodological grounding.

 Suggestion: Explain precisely how IV was computed (binning approach, WoE/IV procedure, thresholds for “high IV”) and add a complementary model-agnostic interpretation check (e.g., permutation importance). If you retain IV as the main interpretability layer, explicitly justify its validity for the models used and discuss limitations.

 5. The manuscript uses many design elements—(i) eight classifiers, (ii) SMOTE balancing, (iii) standardization, (iv) segmentation rules, (v) IV-based early-warning indicators—yet it is not demonstrated which components drive the gains.

 Suggestion: Add ablation experiments such as:

 With vs without SMOTE (or alternative imbalance handling)

 Full feature set vs top-k IV features

 Aggregate modelling vs segmented modelling (show whether segmentation improves AUROC/F1 materially, not just changes IV rankings)

 This will validate the necessity and contribution of each component of the framework.

 6. The manuscript compares multiple ML models and reports multiple metrics but claims about superiority should be supported by statistical comparisons (especially when differences are small for some metrics).

 Suggestion: Add statistical tests for paired model comparisons across folds (e.g., corrected resampled t-test or a nonparametric alternative). Also consider reporting rank-based comparison across folds (average ranks) rather than relying solely on point estimates.

 7. The dataset is described as trade services (wholesale, retail, repair) but acknowledges limited granularity. This heterogeneity could drive model behaviour and variable importance.

 Suggestion: Strengthen the limitation discussion and—if data permits—add a robustness check for listed vs non-listed firms, or at least stratified evaluation to confirm the model is not primarily learning listing status or scale effects. If sub-sector granularity is unavailable, discuss how this may affect generalization and policy recommendations.

 8. Avoid citation of non-peer-reviewed or weakly substantiated works.

 Suggestion: Ensure all references are from peer-reviewed sources. Avoid citing arXiv or preprints papers. Remove or replace them with authoritative journal or conference papers.

 9. While the manuscript cites several strong foundational works on bankruptcy prediction and AI-ML techniques, it omits recent and relevant studies on imbalanced learning, hybrid machine learning strategies, and advanced optimization-based predictive modeling that are directly applicable to financial distress forecasting. The current literature review would benefit from incorporating more recent AI-driven approaches that address class imbalance, hyperparameter optimization, and robust validation in complex predictive settings.

 Suggestion: Incorporate and discuss the following to enrich the literature review and strengthen the methodological positioning of the study:

 Refer to reference 1:

 → Provides direct methodological relevance by addressing bankruptcy forecasting under severe class imbalance conditions using hybrid ML techniques. This study can strengthen the justification for using SMOTE and multiple classifiers in your framework.

 Refer to reference 2:

 → Demonstrates the importance of systematic hyperparameter optimization in improving model robustness and generalization, which is directly relevant to your multi-model comparison strategy.

 Refer to reference 3:

 → Highlights the value of temporal deep learning architectures for financial prediction problems, which can help position your work within broader AI-driven financial forecasting research.

 Refer to reference 4:

 → Illustrates the integration of attention mechanisms and hybrid LSTM architectures to enhance predictive discrimination, offering conceptual support for exploring more advanced deep architectures beyond standard ANN models.

 Incorporating these references would better position your work within the current state-of-the-art in AI-enabled predictive modeling, particularly in handling imbalanced datasets, optimizing model architectures, and strengthening methodological rigor in financial risk forecasting.

 Minor Concerns

 10.  Replace “supervisory machine learning” with “supervised machine learning,” and ensure consistent naming of models/metrics (e.g., recall vs sensitivity).

 11. A careful proofreading pass is recommended to reduce repetition and improve readability.

Is the work clearly and accurately presented and does it cite the current literature?

Partly

If applicable, is the statistical analysis and its interpretation appropriate?

Partly

Are all the source data underlying the results available to ensure full reproducibility?

Partly

Is the study design appropriate and is the work technically sound?

Partly

Are the conclusions drawn adequately supported by the results?

Yes

Are sufficient details of methods and analysis provided to allow replication by others?

Partly

Reviewer Expertise:

machine learning

I confirm that I have read this submission and believe that I have an appropriate level of expertise to confirm that it is of an acceptable scientific standard, however I have significant reservations, as outlined above.

## References

[1] : Advancing Bankruptcy Forecasting With Hybrid Machine Learning Techniques: Insights From an Unbalanced Polish Dataset. *IEEE Access* .2024;12: 10.1109/ACCESS.2024.3354173 9369-9381 10.1109/ACCESS.2024.3354173

[2] : MSPO: A machine learning hyperparameter optimization method for enhanced breast cancer image classification. *DIGITAL HEALTH* .2025;11: 10.1177/20552076251361603 10.1177/20552076251361603 PMC1227755840693252

[3] : Improving Stock Market Predictions: An Equity Forecasting Scanner Using Long Short-Term Memory Method with Dynamic Indicators for Malaysia Stock Market. *Mathematics* .2023;11(11) : 10.3390/math11112470 10.3390/math11112470

[4] : SeizureLSTM: An optimal attention-based trans-LSTM network for epileptic seizure detection using optimal weighted feature integration. *Biomedical Signal Processing and Control* .2024;96: 10.1016/j.bspc.2024.106603 10.1016/j.bspc.2024.106603

# Reviewer report: Reviewer response for version 2

**DOI:** 10.5256/f1000research.194820.r449963

**Published:** 2026-01-24

**Authors:** Vasa László

**Affiliations:** 1 Széchenyi István University, Győr, Hungary

The authors amended the paper accordingly, taking the reviewers' opinions and critics into consideration. I find the paper suitable for indexing in its current for, no additional changes are required.

Is the work clearly and accurately presented and does it cite the current literature?

Partly

If applicable, is the statistical analysis and its interpretation appropriate?

Yes

Are all the source data underlying the results available to ensure full reproducibility?

Yes

Is the study design appropriate and is the work technically sound?

Partly

Are the conclusions drawn adequately supported by the results?

Yes

Are sufficient details of methods and analysis provided to allow replication by others?

Yes

Reviewer Expertise:

economics and management

I confirm that I have read this submission and believe that I have an appropriate level of expertise to confirm that it is of an acceptable scientific standard.

# Reviewer report: Reviewer response for version 1

**DOI:** 10.5256/f1000research.187721.r435705

**Published:** 2025-12-27

**Authors:** Vasa László

**Affiliations:** 1 Széchenyi István University, Győr, Hungary

The research focuses on an actual topic. Predicting bankruptcy in any sector is an evergreen issue, so investigating it in the wholesale, repair and motor vehicle repair looks like an original idea.

 Basically, I like the paper's focus and flow, however, I feel some weaknesses:

 - In the introduction, the reason for this research should be better explained, involving more sources.

 - The literature review is too short, and incomplete; it should be extended significantly, especially involving more latest international sources published in top journals.

 - While I can accept the selected methodology, the proper introduction and explanation is missing, namely why the authors selected the given methodological toolset.

 - the limitations of the research are not highlighted.

 So, I recommend revising the manuscript accordingly.

Is the work clearly and accurately presented and does it cite the current literature?

Partly

If applicable, is the statistical analysis and its interpretation appropriate?

Yes

Are all the source data underlying the results available to ensure full reproducibility?

Yes

Is the study design appropriate and is the work technically sound?

Partly

Are the conclusions drawn adequately supported by the results?

Yes

Are sufficient details of methods and analysis provided to allow replication by others?

Yes

Reviewer Expertise:

economics and management

I confirm that I have read this submission and believe that I have an appropriate level of expertise to state that I do not consider it to be of an acceptable scientific standard, for reasons outlined above.

# Response

**Published:** 2026-01-05

**Authors:** Guruprasad Desai

**Affiliations:** Commerce, Manipal Academy of Higher Education, Manipal, Karnataka, India

1. In the introduction, the reason for this research should be better explained, involving more sources.

**Response:** Thank you for your constructive feedback regarding the need to strengthen the justification and contextual foundation in the introduction. We agree that a more thorough explanation of the research rationale, supported by additional sources, will enhance the manuscript's scholarly contribution and clarity. In response, we will expand the final paragraphs of the introduction to explicitly articulate the research gaps, theoretical motivation, and practical necessity for this study, drawing on relevant literature and empirical evidence.

 2. The literature review is too short, and incomplete; it should be extended significantly, especially involving more latest international sources published in top journals.

**Response:** Thank you for the insightful feedback regarding the literature review. We acknowledge that expanding the review to include a more comprehensive and up-to-date survey of international sources, particularly from top-tier journals, will strengthen the scholarly foundation and contextual relevance of our study. In response, we will significantly extend Section 2: Literature Review by integrating recent (2020–2024) high-impact research on AI/ML-based bankruptcy prediction across sectors and economies, with a specific focus on studies from leading finance, computational intelligence, and risk management journals.

 3. While I can accept the selected methodology, the proper introduction and explanation is missing, namely why the authors selected the given methodological toolset.

**Response:** Thank you for this constructive observation regarding the need for a clearer methodological justification. We agree that a more explicit rationale for the selection of the specific AI-ML models, performance metrics, and analytical framework (including SMOTE and business rules) would strengthen the methodological transparency and scholarly rigor of the paper. In response, we will enhance Section 3: Data and Methodology by adding a dedicated subsection that systematically outlines the reasoning behind each methodological choice, linking them directly to the research objectives, data characteristics, and established practices in the bankruptcy prediction literature.

 4. The limitations of the research are not highlighted.

**Response:** Thank you for this important observation regarding the need to explicitly acknowledge the limitations of our research. We agree that a candid discussion of the study's constraints is essential for scholarly integrity, contextualizes the findings, and provides valuable direction for future research. In response, we will add a dedicated subsection titled "Limitations and Future Research" within the Conclusion section. This will clearly and concisely outline the key limitations pertaining to data, methodology, and generalizability, while simultaneously proposing pathways for subsequent studies to address these constraints.

 Thank you again for this valuable comment. We believe this addition will provide necessary clarity and reinforce the robustness of our research work

# Reviewer report: Reviewer response for version 1

**DOI:** 10.5256/f1000research.187721.r435704

**Published:** 2025-12-24

**Authors:** Tobias Kwame Adukpo, Netifatu Abdulmumin-Butali

**Affiliations:** 1 University for Development Studies, Tamale, Ghana; 2 Information Technology and Management, The University of Texas at Dallas (Ringgold ID: 12335), Texas, USA

The study demonstrates that AI-ML models can predict bankruptcy in India's trade service sector with Random Forest performing best but critical flaws must be fixed:

 1. charity whether SMOTE was applied before/after train-test split to rule out data leakage,

 2. address suspiciously high accuracy rates through external validation or cross validation to prove results are not overfitted and

 3. provide all model hyperparameters and implantation details for replicability.

Is the work clearly and accurately presented and does it cite the current literature?

Partly

If applicable, is the statistical analysis and its interpretation appropriate?

Partly

Are all the source data underlying the results available to ensure full reproducibility?

Yes

Is the study design appropriate and is the work technically sound?

Partly

Are the conclusions drawn adequately supported by the results?

Partly

Are sufficient details of methods and analysis provided to allow replication by others?

Partly

Reviewer Expertise:

Machine learning and deep learning

We confirm that we have read this submission and believe that we have an appropriate level of expertise to confirm that it is of an acceptable scientific standard.

## References

[1] : Impact of Macroeconomic Factors on Government Spending in Ghana. *American Journal of Applied Statistics and Economics* .2025;4(1) : 10.54536/ajase.v4i1.5833 119-126 10.54536/ajase.v4i1.5833

# Response

**Published:** 2026-01-05

**Authors:** Guruprasad Desai

**Affiliations:** Commerce, Manipal Academy of Higher Education, Manipal, Karnataka, India

**Response File**

1. Charity whether SMOTE was applied before/after train-test split to rule out data leakage

Response: Thank you for raising this important methodological point regarding the potential for data leakage in the application of the Synthetic Minority Oversampling Technique (SMOTE). We appreciate the opportunity to clarify this aspect of our procedure and strengthen the manuscript.

 In response to the reviewer's concern, we confirm that in our study, SMOTE was applied exclusively to the training dataset after the train-test split was performed, thereby rigorously preventing any data leakage from the synthetic samples into the test set. The original, unmodified test set was preserved for the final evaluation of all models to ensure an unbiased assessment of their predictive performance on real, unseen data. This approach is a standard and crucial practice to maintain the integrity of model validation when addressing class imbalance.

 We will incorporate this clarification into the revised manuscript to enhance methodological transparency. The most appropriate location for this addition is within Section 3.1 (Data) and Section 3.5 (Class Imbalance – SMOTE Technique), where the data preparation and SMOTE application are described.

 2. Address suspiciously high accuracy rates through external validation or cross validation to prove results are not overfitted

 Response: We thank the respected reviewer for this insightful and important comment regarding the validation of the high accuracy rates reported in our study. In this regard, we have added highlighted the following paragraph along with the other changes here and there in the revised article:

 3.2.1 Model Validation Strategy

 To rigorously evaluate model performance and guard against overfitting, all models were subjected to a repeated k-fold cross-validation procedure. The SMOTE-balanced dataset was randomly partitioned into *k=10* subsets of approximately equal size. For each model, the training and evaluation process was repeated 10 times. In each iteration (or fold), a different subset was used as the hold-out test set, while the remaining nine subsets were combined for training (including the application of SMOTE only on the training fold to prevent data leakage). The performance metrics for each fold were recorded. The final performance metrics presented in the results (Accuracy, Precision, Recall, F1-Score, Specificity, AUROC) represent the mean values calculated across all 10 test folds. This process provides a robust and reliable estimate of the models' predictive generalization capability on unseen data.

 3. Provide all model hyperparameters and implantation details for replicability

 Response: Thank you for the constructive feedback. We appreciate the reviewer’s suggestion to include all model hyperparameters and implementation details to enhance the reproducibility of our study. In response, we will add a new subsection titled “3.2.2 Model Specifications and Hyperparameters” under Section 3.2 (Methodology - AI-ML Models). This subsection will provide a detailed description of the software, libraries, hyperparameter settings, and training procedures used for each of the eight AI-ML models implemented in the study. The addition will ensure that other researchers can replicate our experiments precisely.

 Thank you again for this valuable comment. We believe this addition will provide necessary clarity and reinforce the robustness of our experimental design.
